# Catheter contact area strongly correlates with lesion area in radiofrequency cardiac ablation: an *ex vivo* porcine heart study

**DOI:** 10.1007/s10840-021-01054-3

**Published:** 2021-09-09

**Authors:** Kriengsak Masnok, Nobuo Watanabe

**Affiliations:** 1 Biofluid Science and Engineering Laboratory, Functional Control Systems, Graduate School of Engineering and Science, Shibaura Institute of Technology, Room-102, 6th Building, Omiya-Campus, 307 Fukasaku, Minuma-Ku, Saitama-City, Saitama 337-8570 Japan; 2 Department of Bio-Science and Engineering, College of Systems Engineering and Science, Shibaura Institute of Technology, Saitama, Japan

**Keywords:** Ablation lesion dimensions, Catheter contact area, Catheter contact force, Catheter contact angle

## Abstract

**Purpose:**

Our previous study confirmed that not only force but also the catheter contact angle substantially impacted the contact area and its morphology. Therefore, in this study, we aimed to further investigate the relationship between the catheter contact area and the dimensions of the ablation lesion area as a function of catheter contact angle and force in radiofrequency catheter ablation.

**Methods:**

The radiofrequency catheter ablation test was performed for 5 contact angles and 8 contact forces at a fixed ablation time of 30 s. The initial impedance was 92.5 ± 2.5 Ω, the temperature during ablation was 30 °C, and the power was 30 W. The irrigation rate during ablation was set to 17 mL/min. Each experiment was repeated 6 times.

**Results:**

The catheter contact area showed a strong correlation with the ablation lesion area (*r* = 0.8507). When the contact area was increased, the lesion area also increased linearly in a monotonic manner. The relationships between catheter contact force and ablation lesion area and between catheter contact force and ablation lesion depth are logarithmic functions in which increased contact force was associated with increased lesion area and depth. The catheter contact angle is also an important determinant of the lesion area. The lesion area progressively increased when the contact angle was decreased. In contrast, the lesion depth progressively increased when the contact angle was increased.

**Conclusions:**

The catheter contact area was strongly correlated with the ablation lesion area. Additionally, catheter contact force and contact angle significantly impacted the dimensions of the lesion in radiofrequency catheter ablation procedures.

**Supplementary Information:**

The online version contains supplementary material available at 10.1007/s10840-021-01054-3.

## Introduction

Over the past three decades, radiofrequency (RF) catheter ablation therapy has become a widely used and effective treatment for tachyarrhythmia [1–6]. In RF catheter ablation, the electrical current delivered from the tip electrode of the ablation catheter passes through the contact area of the heart tissue surface and blood. The high alternating current that passes through the resistive tissue generates heat that raises the temperature of the tissue. Once this temperature reaches 50 to 55 °C, the cells in that area coagulate and necrotize [7, 8]. It is well known that the key to safe and effective treatment using RF catheter ablation is to limit the ablation lesion (the area in which necrosis occurs) to a sufficient size without overheating and perforating the surface of the cardiac tissue [9–12].

Given that the catheter contact area is a direct interface between the tip electrode of the ablation catheter and the surface of the heart tissue, we hypothesized that the contact area might be an effective parameter for controlling the dimensions of ablation lesion. In our previous study, we developed an experimental system to investigate the relationship of the catheter contact area on the surface of the heart tissue as a function of catheter contact angle and force. The results confirmed that not only the force but also the catheter contact angle and contact force substantially impacted the contact area and its morphology [13]. Furthermore, we additionally speculated that the catheter’s contact area and contact angle might have a substantial impact on the size of the lesion during ablation therapy.

Consequently, the purpose of this study was to investigate the relationship between the catheter contact area and the dimensions of the ablation lesion as a function of catheter contact angle and force in the radiofrequency catheter ablation process.

## Methods

### Heart muscle surface flattener and preparation

We previously developed an instrument that precisely adjusts the catheter angle between the catheter tip and the heart muscle in order to achieve better reproducibility of *ex vivo* experiments [13]. The instrument comprises a heart muscle surface flattener and catheter tip angle setter. In the heart muscle surface flattener, a circular crystalline acrylic plate with a thickness of 12 mm and a diameter of 130 mm was used to flatten the surface of porcine heart tissue and fix its position and orientation, ensuring that all experiments using this plate will maintain uniformity.

A fresh porcine heart was obtained from a slaughterhouse at 24–48 h after animal sacrifice. A section of the ventricular myocardium was cut into 20–30-mm-thick pieces, and kept at room temperature in a closed container under moist conditions to prevent drying. Before the experiment, the pieces were removed from the closed container and sandwiched between the acrylic plate and a soft sponge placed in a stainless bowl. The surface of a portion of the epicardium lacking adipose tissue was flattened by adjusting the amount of the sponge. The catheter ablation experiments were performed through a hole (20 mm × 50 mm) in the acrylic plate.

### Radiofrequency ablation system incorporating the catheter angle setter and contact force sensor

**Fig. 1:**
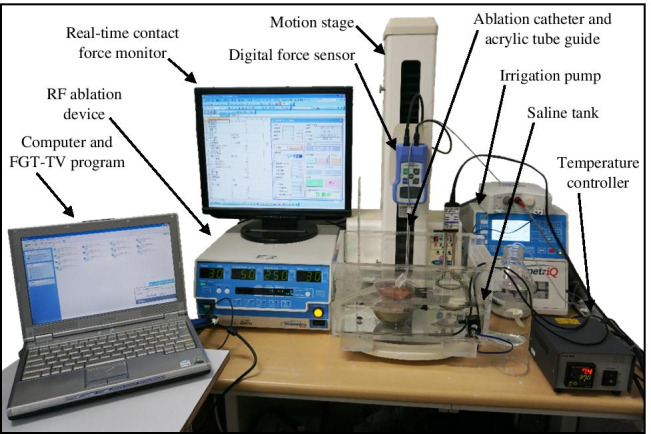
Experimental setup. The saline tank was installed on a compact desktop test stand and equipped with a digital force gauge. The RF ablation device and an irrigation pump were connected to the catheter. The system was operated and monitored using FGT-TV software running on a personal computer

As shown in Fig. 1, a piece of porcine heart prepared using the abovementioned surface flattener was submerged in a tank containing 0.9 wt% saline and the position of the piece was fixed to the bottom of the tank. The position was set by aligning a hole in the acrylic plate with the position of the catheter. The saline tank is equipped with a motion stage having a length of 32 cm, a width of 22 cm, and base that is raised 3 cm from the floor. An anti-slip cover was also installed at the base of the tank. Holes were drilled in two sides of the saline tank to allow for the connection of dispersive electrodes with a screw and a leakproof rubber fitting to provide the return path for the RF current. In the experimental ablation setup, the saline tank was equipped with a motion stage (FGS-5000TV, Nidec-Shimpo Corporation) on which a digital force sensor (FGP-0.5, Nidec-Shimpo Corporation) was mounted. The temperature of the saline solution was maintained at 35 to 37 °C and continuously monitored using a temperature controller (JTA-550, As One Corporation). Circulation flow in the saline-filled chamber was generated by a water pump (AD20P-0510A, DollaTek) to mimic blood flow and distribute temperature. An RF ablation device (Maestro 4000, Boston Scientific Inc.) was used in this study. Saline irrigation with 0.9 wt% was performed using an irrigation pump (MetriQ, Boston Scientific Inc.) that was connected to the catheter. The IntellaNav Mifi™ open-loop irrigated catheter tip (7 Fr/4.5 mm 7.5 Fr; PMR9620, Boston Scientific Inc.) was used in this study. The catheter was 110 cm long, with a tip length of 4.5 mm, and had a standard curve style. The system was operated and monitored using FGT-TV software (Nidec-Shimpo Corporation) running on a personal computer. To investigate the effects of the catheter contact angle and contact force on the ablation dimensions of the heart tissue, we used a procedure that we developed to enable the setting of various catheter contact angles (0, 30, 45, 60, and 90 deg) using a special acrylic tube guide [13]. To set the angle, the catheter was inserted into the tube guide, and the tube guide was locked by turning a screw in the acrylic block mounted on the digital force gauge. The distance between the end-tip of the catheter and the end of the tube guide was fixed by turning the screw.

### Ablation parameters

In the experiments, the 8 levels of contact force within the clinically used range (2, 4, 6, 10, 15, 20, 30, and 40 gf) were applied to the heart tissue surface in line with the typical clinical contact force ranges [14–16]. Using this process, the RF catheter ablation test was repeated 6 times each for the 5 contact angles and 8 contact forces to ensure equal distribution of contact force. The ablation time was fixed at 30 s, and the initial impedance was set at 92.5 ± 2.5 Ω. The temperature during ablation was set at 30 °C, and the power at 30 W. Initially, and before every ablation test, the catheter was placed in the saline tank, where it floated, and an irrigation rate of 2 mL/min (contact force = 0 gf) was set. During ablation, the rate of irrigation with 0.9 wt% saline was subsequently increased from 2 to 17 mL/min [11, 17]. All ablation parameters are shown in Table 1. In the final step, all of the ablation lesion dimensions for each condition were photographed for later evaluation of the ablation lesion dimensions through image analysis. In total, 240 experiments (40 sets of 6 experiments each) were performed.

**Table 1:** Ablation parameters

Parameters	
Ablation time, s	30
Power, W	30
Ablation temperature, °C	30
Saline tank temperature, °C	35–37
Initial irrigation rate, mL/min	2
Ablation irrigation rate, mL/min	17
Catheter contact force, gf	2, 4, 6, 10, 15, 20, 30, and 40
Catheter contact angle, deg	0, 30, 45, 60, and 90

### Evaluation of ablation lesion dimensions and comparison of catheter contact area with ablation lesion area

Conventionally, ablation lesion dimensions are measured using a digital vernier caliper and the lesion area and lesion volume are calculated under the assumption that the ablation lesion is a perfectly symmetrical shape [18–20]. However, in reality, the ablation lesion morphology is never perfectly symmetrical. Moreover, the conventional method requires the investigator to visually estimate the lesion border, which is defined as the location of the change in tissue color. Measuring ablation dimensions in this way may lead to errors in ablation lesion evaluation. In our previous study, we developed an experimental system for investigating the relationship of the catheter contact area on the surface of the heart tissue as a function of catheter contact angle and force. The catheter–tissue contact areas generated using a special visualization technique were described in detail in our previous article [13]. Briefly explaining, special white soluble ink was overlaid on the metal electrode of the catheter tip to visualize the contacted area on the heart tissue surface. Then, the 8 levels of contact force and 5 levels of contact angle were applied to the cardiac muscle. After that, the catheter contact area was photographed, and its contact area morphology on the heart tissue surface was evaluated by using an image analysis program in MATLAB software (version 2021a; The MathWorks, Inc.). The developed system makes possible a new technique for evaluating lesion area as the main aim of our image analysis program was to reduce human error and improve the precision of ablation lesion evaluation. In addition, the primary purpose of the present study is to investigate the relationship between the ablation lesion area and the catheter contact area. Therefore, we used the method for evaluating lesion dimensions and the image analysis program developed in the previous study and then compared each contact area produced in the previous study with the lesion area at the same contact condition (same force, angle, and catheter type).

**Fig. 2:**
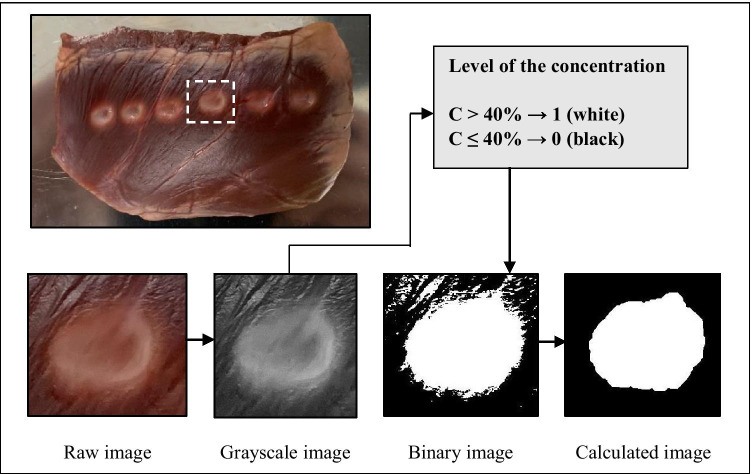
Image analysis process for evaluating the ablation lesion area and its morphology

Immediately after each set of experiments, the 6 ablation lesions were photographed using a camera (Sony A600; lens optical 16–50 mm f/3.5–5.6 OSS) with a reference scale, after which the 6 ablation lesions were bisected along their diameter and photographed again (Fig. 2). The raw image with an image size 24,000,000 pixels was imported to the program and calibrated from the pixel scale to the millimeter scale. Then, the raw image was segmented into individual lesion images and converted into grayscale. The grayscale concentration level was used to define the lesion border at the pixel level, with lesion area defined as white pixels and normal tissue defined as black. In the reversible injury area, the color was not clearly white or black, so we defined the lesion area as that with a 40% concentration of white pixels. Next, each of the pixels was binarized into black or white and the empty area was filled, after which the centroid of each lesion image, the length of the minor and major axes, and lesion region area were calculated. Then, the image of each lesion was rotated about the centroid to make each area’s longest axis parallel to the vertical direction. Lesion depth was measured from the top of the heart tissue surface to the maximum depth and was calculated from 6 experimentally acquired images. The average lesion area and average lesion morphology were also derived from 6 experimentally acquired images.

### Statistical analysis

Pearson’s coefficient (*r*) and Spearman’s coefficient (*r*_s_) were calculated to assess the correlation between each variable. The correlation level was described using Evans’s correlation criterion. Statistical significance was defined as *P* values < 0.05. Comparisons were made using Student’s *t*-test and significant differences were defined as *P* values < 0.05 (95% confidence interval). The coefficient of determination (*R*^2^) was calculated to compare the goodness of fit of the linear and logarithmic models. All statistical analyses were performed using GraphPad Prism (version 9.0.1; GraphPad Software).

## Results

**Fig. 3:**
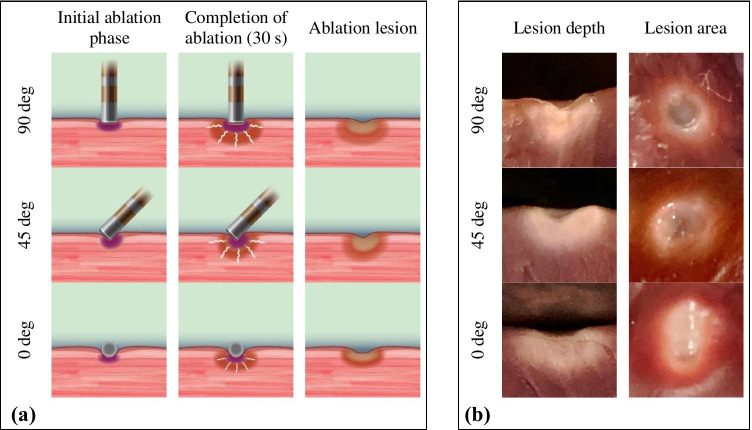
**a** Schematic illustration showing the differences in ablation lesion for each catheter contact angle. **b** Representative examples of lesion depth and lesion area for each contact angle at a contact force of 30 gf

A total of 240 lesions were ablated (40 sets of 6 experiments each); no steam pop events occurred. The data of average lesion area and average lesion depth at each contact angle are shown as means ± SD in Tables 2 and 3, respectively. Figure 3a and b show that not only the catheter contact force but also the catheter contact angle can affect the lesion depth and lesion area. For example, the lesion depth and lesion area differ according to the catheter contact angle, even when the same contact force of 30 gf is applied. Further details about the relationships among catheter contact force, catheter contact angle, catheter contact area, and ablation lesion dimensions will be discussed in the following section.

**Table 2:** Average lesion area (mm^2^)

Contact force	Contact angle				
	0 deg	30 deg	45 deg	60 deg	90 deg
2 gf	16.67 ± 1.63	16.46 ± 3.00	12.93 ± 1.13	9.05 ± 1.79	13.28 ± 1.49
4 gf	24.17 ± 4.14	21.58 ± 2.94	19.98 ± 0.68	19.07 ± 3.43	18.53 ± 1.46
6 gf	24.05 ± 3.46	23.81 ± 1.40	19.76 ± 3.06	20.52 ± 2.23	17.09 ± 3.10
10 gf	35.54 ± 1.59	37.35 ± 3.88	21.70 ± 2.24	20.99 ± 0.79	21.89 ± 1.90
15 gf	37.81 ± 2.87	34.23 ± 3.98	24.01 ± 0.65	23.26 ± 3.55	19.55 ± 0.87
20 gf	36.10 ± 3.18	28.90 ± 6.62	32.59 ± 4.58	30.41 ± 6.69	22.78 ± 3.84
30 gf	38.04 ± 6.07	40.60 ± 6.78	35.37 ± 5.39	33.44 ± 3.79	29.85 ± 1.46
40 gf	44.10 ± 3.50	40.33 ± 6.90	39.38 ± 5.82	41.87 ± 5.68	37.62 ± 7.27

Data are shown as means ± SD

**Table 3:** Average lesion depth (mm)

Contact force	Contact angle				
	0 deg	30 deg	45 deg	60 deg	90 deg
2 gf	1.98 ± 0.30	1.70 ± 0.23	1.92 ± 0.39	2.20 ± 0.40	3.68 ± 0.38
4 gf	1.90 ± 0.14	2.02 ± 0.36	2.14 ± 0.20	2.31 ± 0.32	4.16 ± 0.47
6 gf	2.50 ± 0.32	2.23 ± 0.28	2.49 ± 0.22	3.10 ± 0.66	4.25 ± 0.33
10 gf	3.43 ± 0.53	3.43 ± 0.43	3.04 ± 0.45	3.80 ± 0.45	4.49 ± 0.38
15 gf	3.74 ± 0.40	3.40 ± 0.62	3.46 ± 0.77	4.76 ± 0.65	5.89 ± 0.61
20 gf	4.09 ± 0.43	4.16 ± 0.55	3.78 ± 0.54	4.45 ± 0.56	5.80 ± 0.50
30 gf	4.10 ± 0.21	4.26 ± 0.58	5.56 ± 0.48	5.48 ± 0.60	6.31 ± 0.68
40 gf	4.76 ± 0.36	5.54 ± 0.42	5.75 ± 0.57	5.68 ± 0.47	7.53 ± 0.33

Data are shown as means ± SD

### Relationships between ablation lesion area, catheter contact force, catheter contact angle, and catheter contact area

**Fig. 4:**
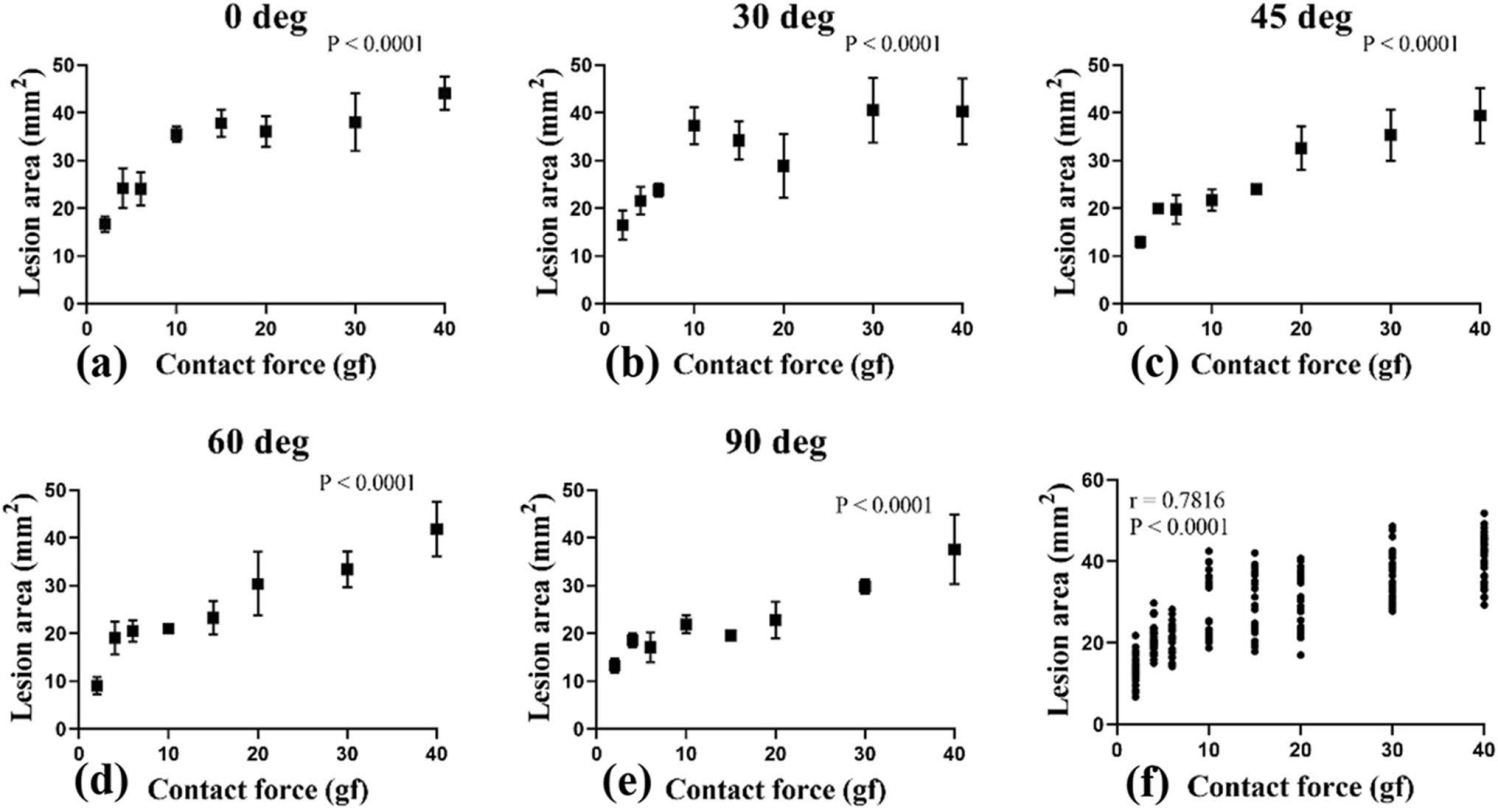
Correlation between catheter contact force and lesion area at contact angle of **a** 0 deg, **b** 30 deg, **c** 45 deg, **d** 60 deg, **e** 90 deg, and **f** all deg

**Fig. 5:**
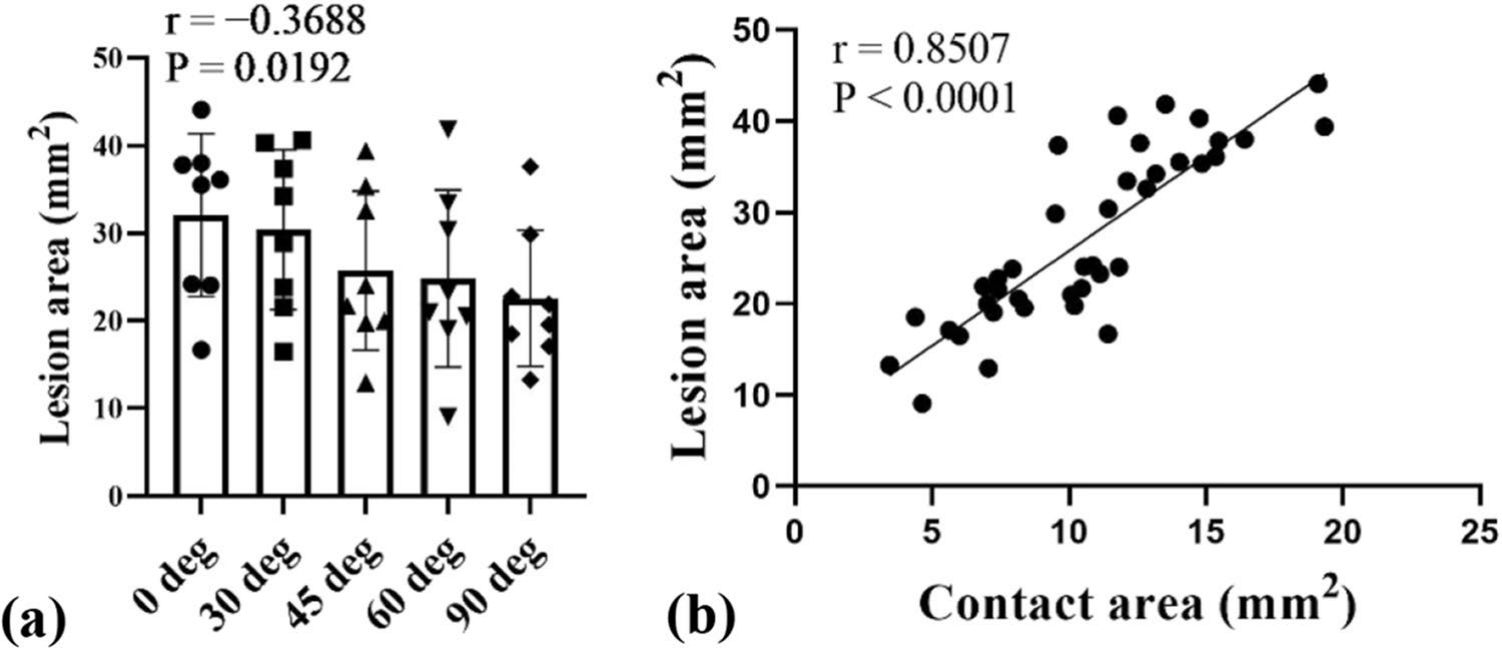
**a** Lesion area as a function of contact force and contact angle. **b** Correlation between the catheter contact area and lesion area

Figure 4 shows the positive correlations (*r* = 0.7816) between catheter contact force (*x*-axis) and lesion area (*y*-axis) at each contact angle. The results revealed that the lesion area increased significantly with increasing contact force (*P* < 0.0001 at every contact angle). Figure 5a is a plot of the correlation between catheter contact angle and lesion area for contact forces ranging from 2 to 40 gf. The results revealed that contact angle is a determinant of lesion area (*r* =  − 0.3688, *P* = 0.0192) (Supplementary Table 1 and Supplementary Fig. 1). The smallest lesion area was produced at a contact angle of 90 deg and increased with decreasing contact angle from 90 to 60, 45, 30, and 0 deg. There were no significant differences in lesion area at a contact angle of 0 vs. 30 deg, 30 vs. 45 deg, 45 vs. 60 deg, and 60 vs. 90 deg (95% confidence interval). However, significant differences were found in lesion area at a contact angle of 0 vs. 45, 60, and 90 deg; 30 vs. 60 and 90 deg; and 45 vs. 90 deg (*P* < 0.05) (Table 4). Figure 5b shows the positive correlation (*r* = 0.8507) between catheter contact area (*x*-axis) and lesion area (*y*-axis). The results revealed that the lesion area increased significantly with increasing contact area (*P* < 0.0001).

**Table 4:** Comparison of lesion area and lesion depth at each contact angle

Contact angle (deg)				Lesion area		Lesion depth
			*P* value	Significantly different (*P* < 0.05)?	*P* value	Significantly different (*P* < 0.05)?
0	vs	30	0.1928	No	0.8186	No
	vs	45	0.0062	Yes	0.4129	No
	vs	60	0.0036	Yes	0.0025	Yes
	vs	90	0.0011	Yes	< 0.0001	Yes
30	vs	45	0.0605	No	0.3735	No
	vs	60	0.0378	Yes	0.0063	Yes
	vs	90	0.0037	Yes	< 0.0001	Yes
45	vs	60	0.2331	No	0.0291	Yes
	vs	90	0.0342	Yes	< 0.0001	Yes
60	vs	90	0.1244	No	< 0.0001	Yes

Table 5 shows the ratio of lesion area to contact area as a function of contact force and contact angle. The ratio of the ablation lesion area to the catheter contact area was calculated using the following equation.

**Table 5:** Ratios of lesion area to contact area at each contact angle

Contact angle	Contact force (gf)							
	2	4	6	10	15	20	30	40
0 deg	1.46	2.23	2.28	2.53	2.45	2.35	2.32	2.31
30 deg	2.74	2.91	3.00	3.89	2.60	1.97	3.45	2.73
45 deg	1.83	2.84	1.94	2.07	2.03	2.54	2.38	2.04
60 deg	1.95	2.63	2.51	2.08	2.09	2.66	2.76	3.10
90 deg	3.86	4.22	3.04	3.18	2.34	3.08	3.14	2.99

$$\mathrm{Ratio of lesion area to contact area }= \left(\frac{\mathrm{Ablated lesion area }}{\mathrm{Catheter contact area}}\right)$$

The results revealed that catheter contact force had no significant relationship with the ratio of lesion area to contact area (*P* = 0.5118) and was only weakly correlated (*r*_s_ = 0.1068). Contact angle had a significant relationship with the ratio of lesion area to contact area (*P* = 0.0175) and was weakly correlated (*r*_s_ = 0.3737) (Supplementary Table 2 and Supplementary Figs. 3, 4, and 5).

### Relationships between catheter contact force, catheter contact angle, and ablation lesion depth

**Fig. 6:**
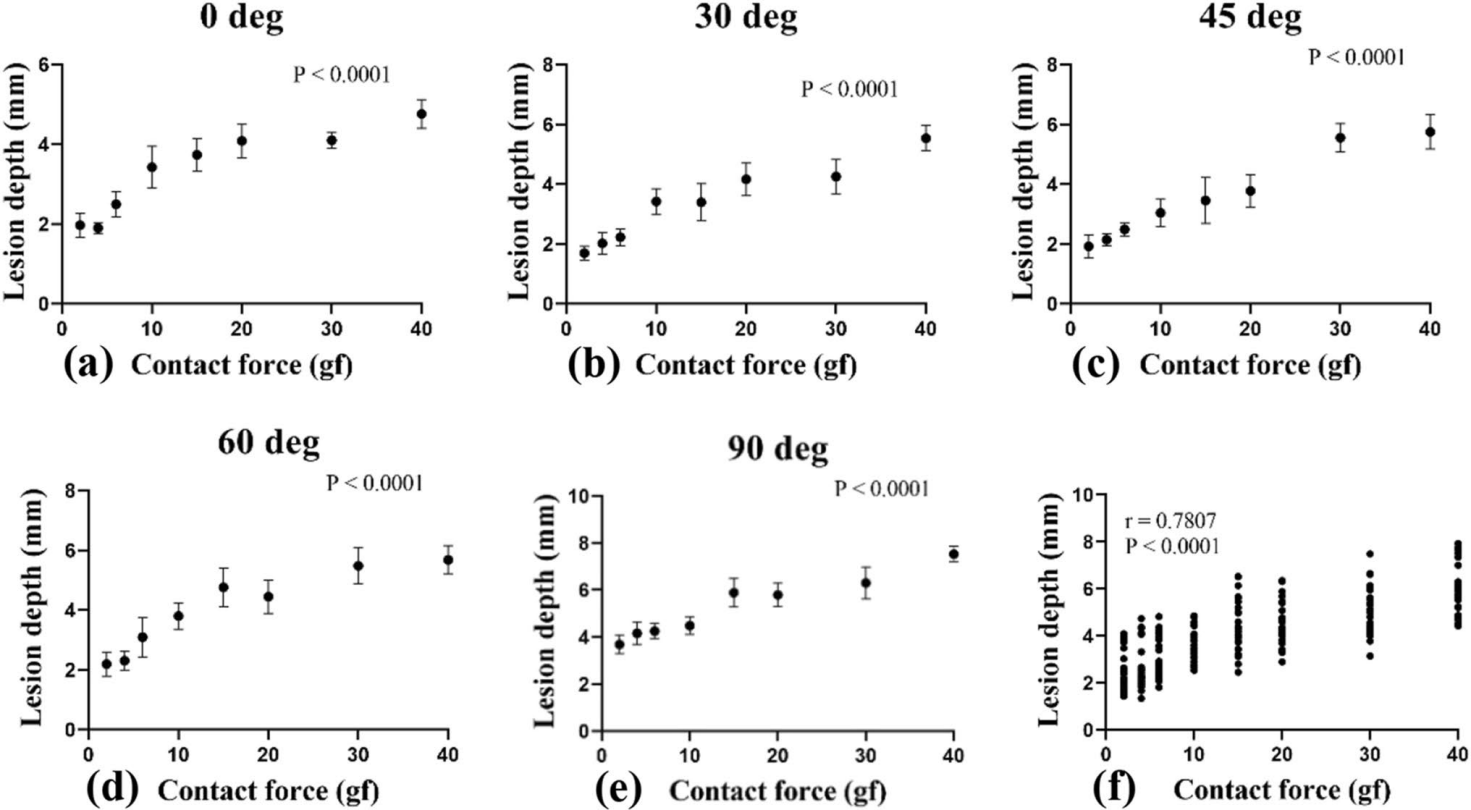
Correlation between catheter contact force and lesion depth at contact angle of **a** 0 deg, **b** 30 deg, **c** 45 deg, **d** 60 deg, **e** 90 deg, and **f** all deg

**Fig. 7:**
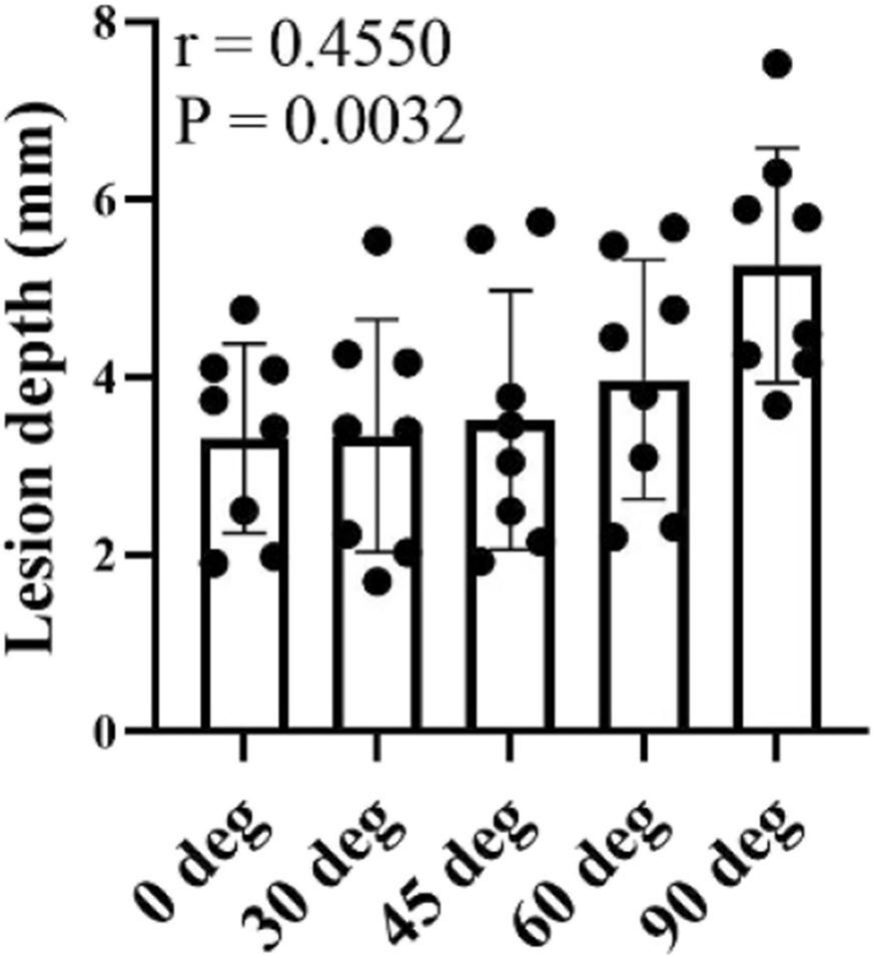
Lesion depth as a function of contact force and contact angle

Figure 6 shows the positive correlations (*r* = 0.7807) between catheter contact force (*x*-axis) and lesion depth (*y*-axis) at each contact angle. The results revealed that lesion depth increased significantly with increasing contact force (*P* < 0.0001 at every contact angle). Figure 7 is a plot of the correlation between catheter contact angle and lesion area for contact forces ranging from 2 to 40 gf. The results revealed that contact angle is a determinant of lesion depth (*r* = 0.4550, *P* = 0.0032) (Supplementary Table 1 and Supplementary Fig. 2). The smallest lesion depth was produced at a contact angle of 0 deg and increased with increasing contact angles from 0 to 30, 45, 60, and 90 deg. There were no significant differences in lesion depth at a contact angle of 0 vs. 30 deg, 0 vs. 45, and 30 vs. 45 (95% confidence interval). However, significant differences were found in lesion depth at a contact angle of 0 vs. 60 and 90 deg; 30 vs. 60 and 90 deg; 45 vs. 60 and 90 deg; and 60 vs. 90 deg (*P* < 0.05) (Table 4). Further details will be discussed below in the Sect. 4.

## Discussion

### Major findings

Our major findings are as follows. First, the catheter contact area showed a strong correlation with the ablation lesion area. When the contact area was increased, the lesion area also increased linearly in a monotonic manner. Second, the relationships between catheter contact force and ablation lesion area and between catheter contact force and ablation lesion depth are logarithmic functions in which increased contact force was associated with increased lesion area and depth. Third, the catheter contact angle is also an important determinant of the lesion area. The lesion area progressively increased when the contact angle was decreased. In contrast, the lesion depth progressively increased when the contact angle was increased.

### Correlation between catheter contact area and ablation lesion area

The catheter–tissue contact area is a direct interface between the tip electrode of the ablation catheter and the surface of the heart tissue and depends on the contact conditions resulting from the combination of the contact force and contact angle. The electrical current delivered from the tip electrode of the ablation catheter passes through the contact area of the heart tissue surface and generates heat that raises the temperature of the tissue, causing the cells in that area to necrotize. Accordingly, the catheter contact area is an important consideration when planning ablation procedures. Unfortunately, the attention this parameter receives does not match its importance. A possible reason for this may be the difficulty of visualizing the catheter–tissue contact area, which we successfully achieved in our previous study [13]. We speculated that the catheter contact area as a function of catheter contact force and catheter contact angle might substantially impact the size of the ablation lesion. We additionally hypothesized that the catheter contact area and lesion area morphology might be similar.

The results of the present study confirmed our hypotheses by revealing a very strong correlation between the catheter contact area and the ablation lesion area, as shown in Table 6. When the contact area was increased, the lesion area also increased. The relationship between catheter contact area and lesion area as a function of catheter contact angle and force can be summarized and expressed as the simple linear regression approximation formulas shown in Table 7. We also found that the lesion area morphology was almost the same as the catheter contact area. The lesion area morphology can be divided into three shapes: oval, circle, and ellipse, as shown in Fig. 3b. In addition, we calculated the ratio of lesion area to contact area as a function of contact force and contact angle, as shown in Table 5. The results showed that catheter contact force had no significant relationship with the ratio of lesion area to contact area, whereas the contact angle did. Both also showed a very weak correlation with the ratio of lesion area to contact area. The contact area at a contact angle of 90 deg had the largest ratio compared with the same contact force at other contact angles. These data might describe the possible contact area at each angle and its relationship to the resulting lesion area. However, it should be noted that this amount of information may not be sufficient to conclude the exact contact area given that other factors were not considered.

**Table 6:** Correlation level and direction trend between each factor

	Pearson’s coefficient (*r*)*	Correlation level**
Contact force vs. lesion area	( +) 0.7816	Strong
Contact angle vs. lesion area	( −) 0.3688	Weak
Contact area vs. lesion area	( +) 0.8507	Very strong
Contact force vs. lesion depth	( +) 0.7807	Strong
Contact angle vs. lesion depth	( +) 0.4550	Moderate

*Positive values ( +) denote positive correlations and negative values ( −) denote negative correlations

**Correlation level based on the absolute value of *r*: 0.00–0.19 is very weak, 0.20–0.39 is weak, 0.40–0.59 is moderate, 0.60–0.79 is strong, 0.80–1.0 is very strong, and a value of 0 denotes no correlation

**Table 7:** Approximation formulas expressing the relationship between catheter contact area and ablation area as a function of contact force for each catheter contact angle

Angle (deg)	Lesion area approximation formula	*R*^2^	Contact area approximation formula	*R*^2^
0	*Y* = 2.413*X* − 0.8227	0.9653	*X* = 2.685ln(*Z*) + 7.782	0.837
30	*Y* = 2.723*X* + 1.791	0.8799	*X* = 3.036ln(*Z*) + 3.465	0.845
45	*Y* = 2.079*X* + 1.395	0.8717	*X* = 3.689ln(*Z*) + 2.952	0.867
60	*Y* = 3.232*X* – 6.825	0.8709	*X* = 2.807ln(*Z*) + 3.137	0.984
90	*Y* = 2.601*X* + 4.486	0.9691	*X* = 2.693ln(*Z*) + 0.892	0.893

*X* is the catheter contact area (mm^2^), *Y* is the ablation lesion area (mm^2^), *Z* is the catheter contact force (gf), and *R*^2^ is the coefficient of determination (for 2 ≤ Z ≤ 40)

### Correlation between catheter contact force, ablation lesion area, and lesion depth

The catheter contact force showed a strongly positive correlation with ablation lesion area and depth, as shown in Table 6. When the contact force was increased, the lesion area and depth also increased. However, it is essential to consider the small changes in lesion area and depth that occurred at higher contact forces. As shown in Figs. 4 and 6, the slope of the graph changes slightly when the contact force is between 15 and 40 gf and increases more during initial contact, when the contact force ranges from 2 to 15 gf. To clarify the behavior of the correlations among ablation lesion area, lesion depth, and catheter contact force, goodness of fit was calculated to facilitate comparison (Table 8; Supplementary Fig. 6). The coefficient of determination (*R*^2^) of each condition revealed that the lesion area increased monotonically but logarithmically at contact angles of 0, 30, 45, and 60 deg but not 90 deg. The lesion depth also increased logarithmically at contact angles of 0, 30, and 60 deg but not 45 and 90 deg. However, it should be noted that there was a slight difference in the value of *R*^2^ under each condition. The results of our study are not surprising; prior studies of *ex vivo* experimental models have also found a similar tendency for increasing catheter contact force to correlate with increasing lesion area and depth. Yokoyama et al. [11] performed irrigated-tip ablation at contact forces of 2, 10, 20, 30, and 40 gf using a canine thigh model. They found that increasing contact force was significantly associated with larger lesions. They concluded that the effect of catheter contact force was a more important determinant of lesion size compared with the delivered power. Thiagalingam et al. [21] also confirmed the importance of catheter contact force during irrigated ablation by using 3 different contact forces (2, 20, and 60 gf). They also concluded that catheter contact force has an important impact on ablation lesion size. Some evidence from *in vivo* studies and human studies have also shown the same tendency [10, 14, 16, 22].

**Table 8:** Comparison (*R*^2^) between the logarithmic and linear fit of the catheter contact force with lesion area and lesion depth at each contact angle

Contact angle (deg)	Contact force vs. lesion area			Contact force vs. lesion depth		
	Logarithmic (*R*^2^)	Linear (*R*^2^)	Behavior of the correlation	Logarithmic (*R*^2^)	Linear (*R*^2^)	Behavior of the correlation
0	0.7828	0.6484	Log	0.8481	0.7538	Log
30	0.6203	0.5181	Log	0.8573	0.8256	Log
45	0.8392	0.8107	Log	0.8636	0.8761	Linear
60	0.8161	0.8000	Log	0.8355	0.7524	Log
90	0.7232	0.7921	Linear	0.8280	0.8377	Linear

### Correlation between catheter contact angle, ablation lesion area, and lesion depth

Catheter contact angle is a determinant of lesion area and depth, as shown in Table 6. However, the catheter contact angle and ablation lesion area are only weakly correlated. The lesion area progressively increased when the contact angle was decreased, as shown in Fig. 5a. The smallest lesion area was produced at a contact angle of 90 deg and increased with decreasing contact angle from 90 to 60, 45, 30, and 0 deg. The catheter contact angle and ablation lesion depth were moderately correlated. The lesion depth progressively increased when the contact angle was increased, as shown in Fig. 7. The smallest lesion depth was produced at a contact angle of 0 deg and increased with increasing contact angle from 0 to 30, 45, 60, and 90 deg.

The catheter contact angle plays another role in lesion area morphology, as shown in Fig. 3b. The lesion area morphology is oval (egg-like) when contact is made at an oblique catheter orientation (30, 45, and 60 deg). However, when contact is made at a parallel catheter orientation (0 deg), the lesion area morphology is elliptical, whereas a perpendicular catheter orientation (90 deg) created a circular lesion area. Kawaji et al. [19] reported very similar results. They evaluated the lesion size in porcine hearts with an 8-Fr open-tip irrigated catheter at 3 different contact angles (0, 45, and 90 deg), 3 levels of power (25, 30, and 35 W), and 3 contact forces (5, 15, and 30 gf). In their report, oblique and parallel catheter orientations created an oval lesion area, whereas the perpendicular catheter orientation created a circular lesion area. In addition, they also concluded that the lesion depth significantly increased with a perpendicular rather than parallel orientation, but the lesion volume did not show a significant difference. Iwakawa et al. [18] also reported a similar tendency. They reported that a parallel catheter orientation created a significantly larger lesion area and a comparatively shallower lesion depth. Chan et al. [23] confirmed that the catheter orientation had a more pronounced effect on lesion dimensions compared with tip size alone. Lesions became larger with each increment in catheter tip length when the tip electrode was positioned parallel to the tissue surface. Calzolari et al. [24] also demonstrated that catheter contact angle plays a significant role in lesion size, but drew different conclusions. They used in vitro experimental model to create ablation lesions on a porcine heart with a fixed contact force of 20 gf at contact angles of 0, 45, and 90 deg. They concluded that the superficial lesion length increased as the catheter shifted from a perpendicular to a parallel orientation. The absolute maximal lesion length was greater with an oblique catheter orientation. However, their results showed that the lesion width was similar regardless of the orientation. This discrepancy between their findings and ours might be due to different experimental settings. In their study, the contact force was fixed at 20 gf, but we used various catheter contact forces ranging from 2 to 40 gf. In addition, differences in the catheter platform, including the shape of the catheter tip and irrigation rate, might also be a factor. Therefore, the effects of catheter contact angle on lesion dimensions require further investigation in order to provide sufficient knowledge that can be applied in the clinical setting.

## Clinical implications

Precise control of lesion dimensions is an essential parameter for treatment strategies. Catheter contact area might be another effective parameter for controlling the ablation lesion dimensions, given that the catheter contact area is a direct interface between the tip electrode of the ablation catheter and the surface of the heart tissue. However, the data obtained in this study cannot be applied directly to clinical practice on a beating human heart, especially in terms of lesion size. Nevertheless, the findings of our study support a possible role of catheter contact area imaging for assessing ablation lesion dimension. This study provides data showing a very strong correlation between catheter contact area and ablation lesion area. It also provides approximation formulas for estimating lesion area as a function of contact area and contact force for each contact angle. Our data should help clinicians performing this procedure to understand the relationships among the parameters and plan their experiment strategy accordingly. Lastly, our data suggest that the extent to which lesion size can be increased by increasing the contact force may be limited. The catheter contact angle relative to the surface of the heart muscle tissue should also be considered when calculating the desired lesion size.

## Study limitations

This study has several limitations. First, our study was conducted using an *ex vivo* model consisting of a porcine heart, so there was no respiratory motion, catheter instability, or cardiac beating. However, because a precisely controlled model was required to achieve the purpose of this study, the *ex vivo* model was deemed appropriate for this study. In addition, the experimental heart model did not include coronary perfusion and used saline instead of blood (saline has a higher electrical conductivity compared with blood). Therefore, the results cannot be applied directly to clinical practice on a beating human heart, especially in terms of lesion size. Second, the instrument used in this study features a heart muscle surface flattener and a catheter tip angle setter in order to ensure reproducibility. However, in clinical practice, the shape of the heart tissue surface varies according to the part of the heart, and thus, the catheter tip orientation can rarely be optimized due to the various anatomical structures of the heart. Nevertheless, to achieve the purpose of this study, it was necessary to perform tests on flat surfaces to clearly show the effects of the investigated parameters on the surface of the heart tissue. Third, our study used an open-loop irrigated catheter tip, specifically a “flat-tip catheter,” with a pre-determined size and width. Thus, these results might not be reproducible with other commercially available catheters. Lastly, the approximation formulas for estimating contact and lesion area are limited to procedures using the same ablation parameters as in this study. However, our results revealed interesting relationships among the parameters as well as the effect of catheter contact force and contact angle on the contact area and lesion dimensions. Thus, our findings should be further investigated by conducting *in vivo* experiments, animal model experiments, or studies based on practical clinical treatment.

## Conclusion

This study revealed a strongly significant positive correlation between catheter contact area and ablation lesion area. The findings clearly demonstrated a substantial impact of catheter contact force, contact angle, and contact area on lesion dimensions in RF catheter ablation procedures. Such information should be helpful in the selection of effective values for contact force and contact angle in order to predict lesion size as well as for clinicians performing this procedure to understand the relationships among the parameters and plan their ablation strategy accordingly.

## Supplementary Information

Below is the link to the electronic supplementary material.

Supplementary file1 (DOCX 666 KB)

## Data Availability

The datasets generated and/or analyzed during the current study are available from the corresponding author upon reasonable request.

## Code availability

MATLAB (Version 2021a) and GraphPad Prism software (Version 9.0.1) were used in this study. Code generated and/or analyzed during the current study are available from the corresponding author upon reasonable request.
