# Supplementary material for: Catheter contact area strongly correlates with lesion area in radiofrequency cardiac ablation: an ex vivo porcine heart study
Source: J Interv Card Electrophysiol. 2021 Sep 9;63(3):561–72. doi: 10.1007/s10840-021-01054-3 (PMC9151538; doi:10.1007/s10840-021-01054-3)
Supplement: Supplementary file 1 — Supplementary file1 (DOCX 666 KB) [file 10840_2021_1054_MOESM1_ESM.docx]

**SUPPLEMENTAL MATERIAL**

**Manuscript ID:** JICE-D-21-00153

**Journal:** *Journal of Interventional Cardiac Electrophysiology*

**Authors:** Kriengsak Masnok and Nobuo Watanabe

**Corresponding author:** Nobuo Watanabe

Email: [nobuo@sic.shibaura-it.ac.jp](mailto:nobuo@sic.shibaura-it.ac.jp)

ORCID: 0000-0001-9753-898X

These materials are intended for publication as a data supplement.

**Supplementary Table 1** Correlation between contact angle and lesion area and depth.

|  | Contact angle vs. lesion area | Contact angle vs. lesion depth |
| --- | --- | --- |
| Pearson’s r | −0.3688 | 0.4550 |
| 95% confidence interval | −0.6102 to −0.06470 | 0.1672 to 0.6714 |
| P (two-tailed) | 0.0192 | 0.0032 |
| Significant? (alpha = 0.05) | Yes | Yes |


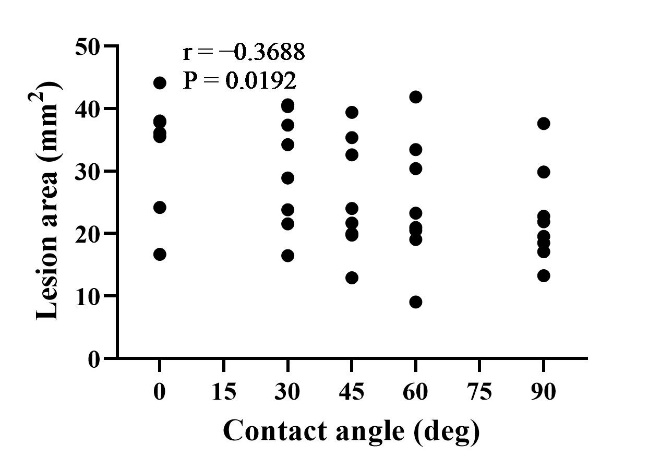


**Supplementary Fig. 1.** Lesion area as a function of contact force and contact angle.


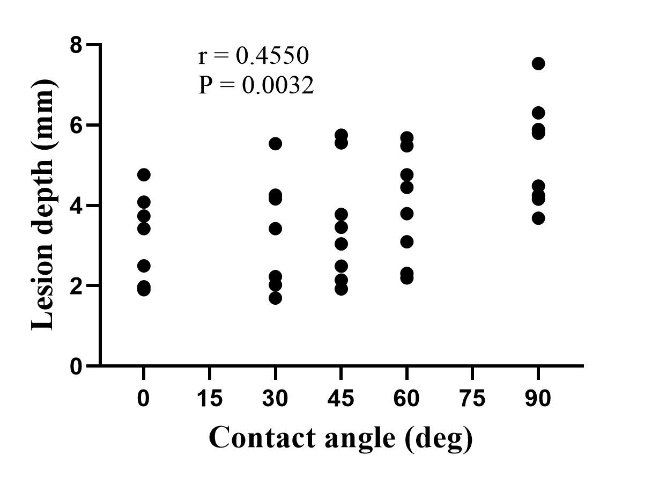


**Supplementary Fig. 2** Lesion depth as a function of contact force and contact angle.

**Supplementary Table 2** Correlation of contact force and contact angle with the ratio of lesion area to catheter contact area.

|  | Contact force vs. lesion area/contact area | Contact angle vs. lesion area/contact area |
| --- | --- | --- |
| Spearman r_s_ | 0.1068 | 0.3737 |
| 95% confidence interval | −0.2208 to 0.4128 | 0.06086 to 0.6196 |
| P (two-tailed) | 0.5118 | 0.0175 |
| Significant? (alpha = 0.05) | No | Yes |


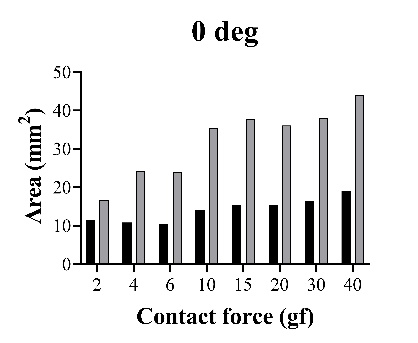

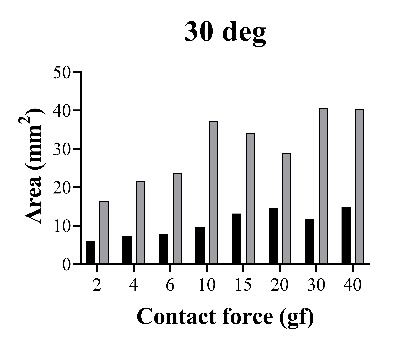

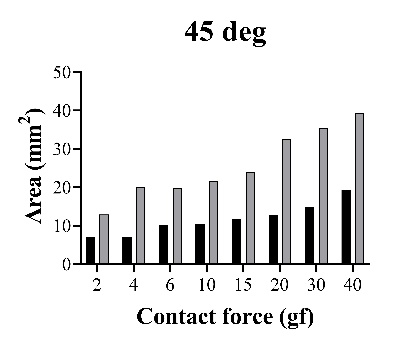

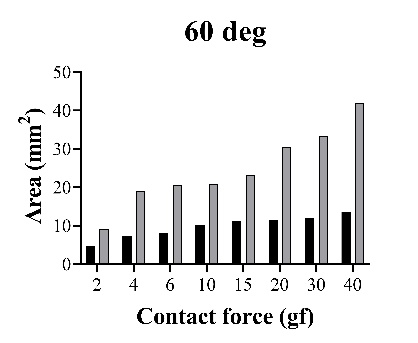

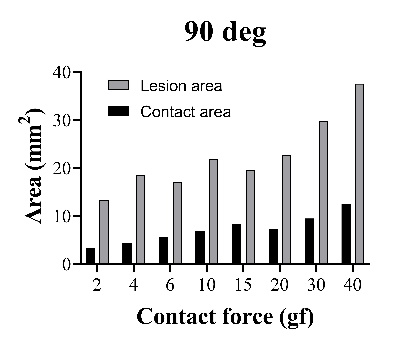


**Supplementary Fig. 3** Comparison of the ratio of lesion area to catheter contact area at each contact angle.


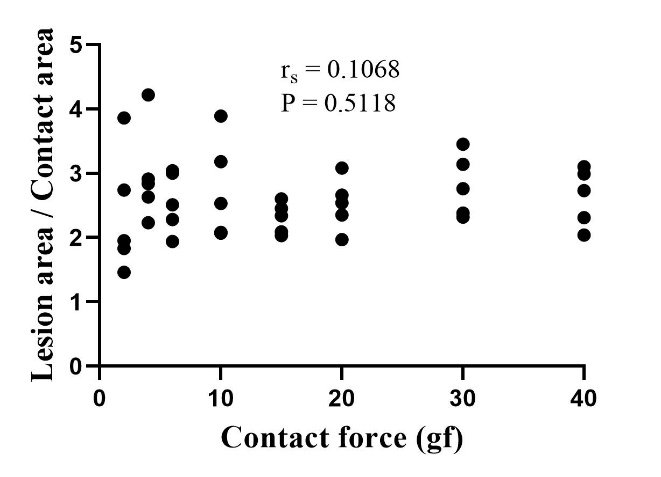


**Supplementary Fig. 4** Correlation between catheter contact force and the ratio of lesion area to catheter contact area.


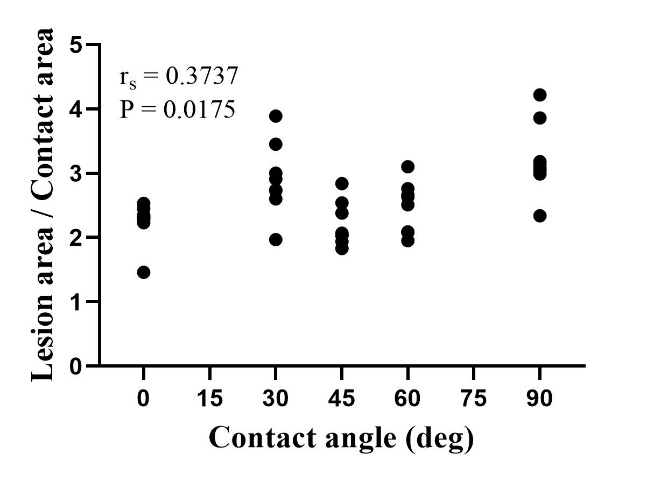


**Supplementary Fig. 5** Correlation between catheter contact angle and the ratio of lesion area to catheter contact area.

| 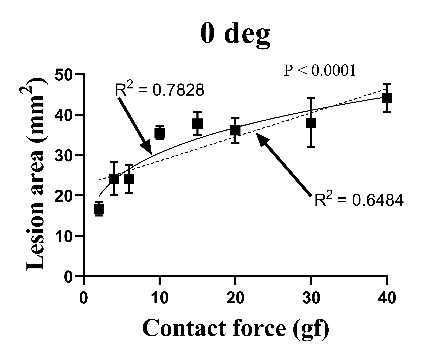 | 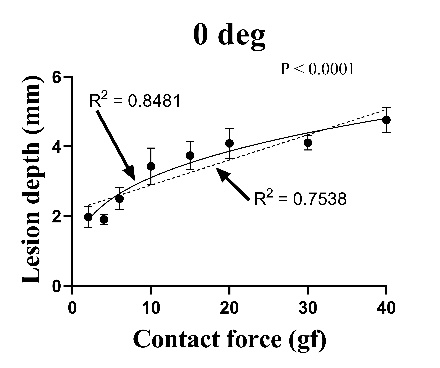 |
| --- | --- |
| 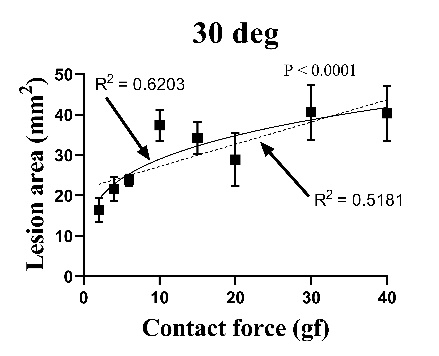 | 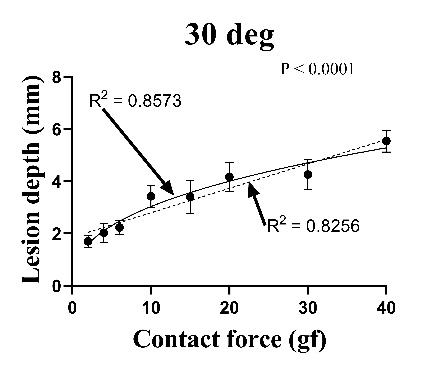 |
| 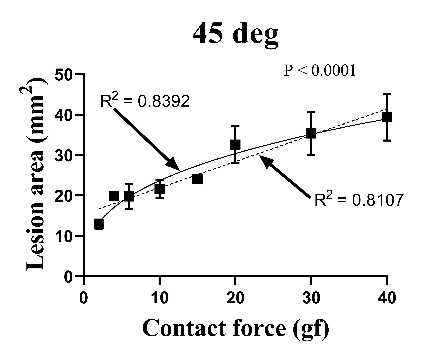 | 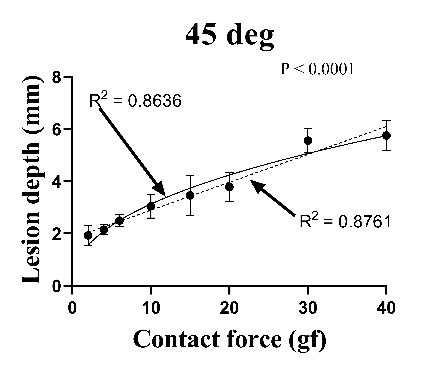 |
| 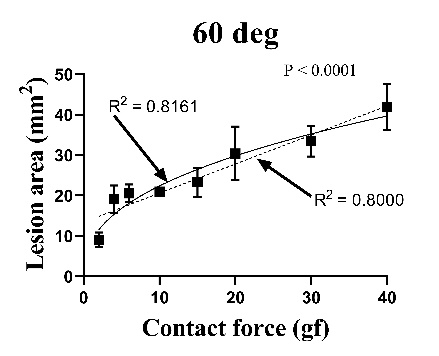 | 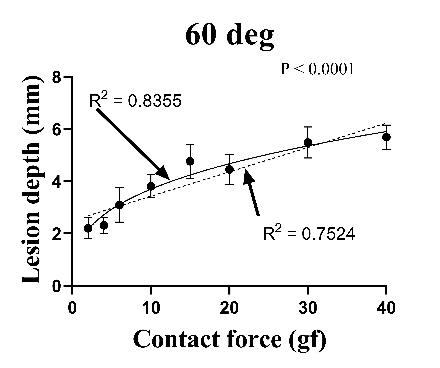 |
| 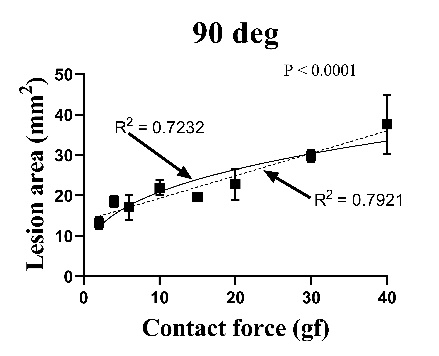  **(a)** | 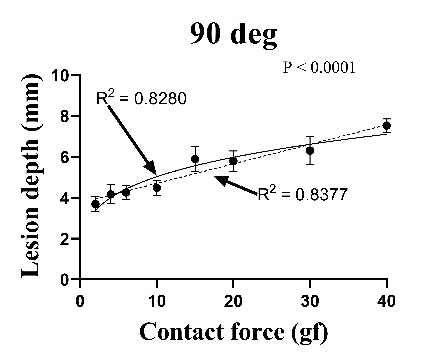  **(b)** |

**Supplementary Fig. 6 (a)** Comparison (R^2^) of the logarithmic and linear fit of the catheter contact angle with the lesion area; **(b)** Comparison (R^2^) of the logarithmic and linear fit of the catheter contact angle with the lesion depth.
